# A living biobank of matched pairs of patient-derived xenografts and organoids for cancer pharmacology

**DOI:** 10.1371/journal.pone.0279821

**Published:** 2023-01-05

**Authors:** Xiaoxi Xu, Rajendra Kumari, Jun Zhou, Jing Chen, Binchen Mao, Jingjing Wang, Meiling Zheng, Xiaolong Tu, Xiaoyu An, Xiaobo Chen, Likun Zhang, Xiaoli Tian, Haojie Wang, Xin Dong, Zhengzheng Bao, Sheng Guo, Xuesong Ouyang, Limei Shang, Fei Wang, Xuefei Yan, Rui Zhang, Robert G. J. Vries, Hans Clevers, Qi-Xiang Li

**Affiliations:** 1 Crown Bioscience Inc., Beijing, China; 2 Crown Bioscience Inc., San Diego, California, United States of America; 3 Crown Bioscience Inc., Taicang City, Jiangsu, China; 4 Shanghai Yihao Biological Technology, Xuhui District, Shanghai, China; 5 Suzhou NeoLogics Bioscience Co, LTD, Suzhou, China; 6 Hubrecht Organoid Technology (HUB), Utrecht, The Netherlands; 7 Oncode Institute, Hubrecht Institute, Royal Netherlands Academy of Arts and Sciences and University Medical Center, Utrecht, The Netherlands; Bauer Research Foundation, UNITED STATES

## Abstract

Patient-derived tumor xenograft (PDX)/organoid (PDO), driven by cancer stem cells (CSC), are considered the most predictive models for translational oncology. Large PDX collections reflective of patient populations have been created and used extensively to test various investigational therapies, including population-trials as surrogate subjects *in vivo*. PDOs are recognized as *in vitro* surrogates for patients amenable for high-throughput screening (HTS). We have built a biobank of carcinoma PDX-derived organoids (PDXOs) by converting an existing PDX library and confirmed high degree of similarities between PDXOs and parental PDXs in genomics, histopathology and pharmacology, suggesting “biological equivalence or interchangeability” between the two. Here we demonstrate the applications of PDXO biobank for HTS “matrix” screening for both lead compounds and indications, immune cell co-cultures for immune-therapies and engineering enables *in vitro/in vivo* imaging. This large biobank of >550 matched pairs of PDXs/PDXOs across different cancers could become powerful tools for the future cancer drug discovery.

## Introduction

The drug discovery/development workflow has been rather standardized and practiced over the decades, encompassing a linear, long (10~15 years) and costly (> $1 billion) process with high attrition rates [1]. Oncology drug development attrition is higher than many other therapeutic areas, with only 3.4%~5% success in clinical development and many agents failing in phase II studies [1, 2]. One cause for the high failure rate is the large gap between preclinical and clinical validation. Cancer is not a single, but a complex disease, per histopathology, genomics, heterogeneous cancer cells (CSC-and differentiation), and immune-diversity and thus preclinical observations do not sufficiently predict or translate into clinical results. In addition, successful cancer drug development requires rapid identification of not only potent drug leads, but also precise patient populations, rendering the current linear workflow ineffective and unproductive. An alternative approach would consist of a “matrix” screen, which simultaneously screens for large numbers of models (for indications) or compounds (for leads), at the early stages of drug discovery/development, and which would be more rapid, risk-adverse and cost-effective.

Patient-derived xenografts (PDXs) [3], or libraries of PDXs [4, 5], have long been viewed as the model of choice to test chemo- and/or targeted therapies, closely mimicking human disease and heterogenous patient populations, although less adequate to test immune-oncology (IO) strategies for their immune-compromised nature [6]. PDXs are cancer stem cell (CSC)-based disease models, featuring genetic stability, in contrast to cell line-based xenografts. Over the last 15 years, tens of thousands of PDXs have been established globally [4, 5, 7], becoming a workhorse for cancer drug evaluation in the preclinical setting [4]. Our laboratory has established a large library of PDXs covering different cancer types, including many of epithelial origins, *e*.*g*. lung [7, 8], colorectal [9], gastric [10], and esophageal [11] cancers. Many PDXs have been extensively tested for various investigational therapies with great translational value [12–14], including those in the format of population-based trials (mouse clinical trial) [9–11, 15, 16], enabling the selection of the “right” patient populations for downstream clinical development.

Although widely used, PDX models are intrinsically costly, time consuming with low throughput, inadequate for large scale screening, as well as being encumbered with ethical/regulatory limitations. It is thus a practical challenge to take advantage of the already available large PDX libraries. In addition, there are other limitations of PDX as a platform to meet the demand of today’s pharmaceutical research. PDX can only be used for limited IO research, except for those exogeneous cell therapies, *e*.*g*. chimeric antigen receptor T (CAR-T) cells [6, 12]. Humanized mice engrafted with human immunity are by no means satisfactory due to a lack of adequate immunity or the severe toxicity caused by graft versus host disease (GvHD). It is also difficult to engineer PDXs through gene-editing for different applications, including orthotopic imaging, introduction of resistant mutations, validation of specific targets [17], *etc*.

The recent establishment of *in vitro* patient-derived organoid (PDOs) cultures of epithelial origins based on tissue-committed adult stem cells [18], including malignant organoids driven by CSC of different carcinomas [19–26], have been shown to be genetically-stable [23, 27] and highly predictive of clinical treatment outcomes [24, 28–30]. In addition, the *in vitro* setting of PDO enables high-throughput screening (HTS) [31], which can thus potentially take advantage of the available large libraries of patient-derived surrogate models allowing a matrix-screen, thus overcoming the limitations of PDXs.

Here we converted our existing large PDX libraries [5] into organoids (PDX-derived organoids, or PDXOs), thus creating a large cancer organoid biobank quickly and enabling HTS, as well as creating a unique, matched patient-derived disease libraries of *in vivo* and *in vitro* models. This report also describes the characterization, in the context of comparing PDXO to their parental PDXs, for genomics, histopathology and pharmacology. Our data reveals that the paired PDX/PDXO are biologically equivalent/interchangeable with various applications in drug discovery.

## Materials and methods

### PDX processing

PDX establishment, maintenance and biobanking have been previously described [7], including models being individually quality controlled and tracked (authentication and virus infection status and mycoplasma status *etc*.) [32]. Briefly, all PDX were maintained in immunocompromised mice. When tumor volume (TV) reached 500–700 mm^3^ (1/2 length x width^2^), tumor tissue (excluding necrotic, calcified and normal tissue) was harvested for either engraftment into mice for pharmacology studies or dissociated for use in 3D *ex vivo* assays (S4 Fig) or organoid generation. All the protocols and amendment(s) or procedures involving the care and use of animals were reviewed and approved by the Crown Bioscience Institutional Animal Care and Use Committee (IACUC) prior to conducting the studies. The care and use of animals was conducted in accordance with AAALAC (Association for Assessment and Accreditation of Laboratory Animal Care) International guidelines as reported in the Guide for the Care and Use of Laboratory Animals, National Research Council (2011). All animal experimental procedures were under sterile conditions at SPF (specific pathogen-free) facilities and conducted in strict accordance with the Guide for the Care and Use of Laboratory Animals from the National Institute of Health, AVMA (2020) and ARRIVE guidelines [33].

### Generation of PDXO biobank

The method for establishment of PDXOs from PDXs or cryopreserved PDX tumor fragments has been previously described [11, 23, 26, 31, 34–39] to create organoid cultures. Generally, fresh PDX tissue if easily available was more successful for organoid establishment, however cryopreserved tumor fragments reduced the requirement for establishing xenograft first. Briefly, tumor tissue from each cancer type were minced, digested by Collagen B for 20 to 60 minutes, depending on the digesting progress. After digesting, the tissue fragment suspensions were washed through a 100 μm cell strainer. Pass through were washed twice and put on ice. Pre-cooled Matrigel^®^ were used to mix with fragment suspension to make a concentration of 70%. Droplets of Matrigel suspension were solidified at 37°C in 6 well plates and organoid culture media added to each well and incubated at 37°C and 5% CO_2_. Growth and morphology of organoids was assessed by bright field microscopy. Organoid culture medium was refreshed every 3–4 days and organoids passaged every 7–14 days at certain ratios depending on the density of the culture and models and transferred to 24 well plates. The expanded organoids culture were cryopreserved to create a master biobank of early passage organoids [31, 36], which were quality controlled and tracked similar to our PDX biobank [32, 40].

### Histopathology (H&E), IHC and IF analysis

PDXO characterization by histopathology has been previously described [36]. Briefly, organoids were collected from the culture well, followed by centrifugation, washed with PBS and fixed in 10% formalin for 1 hour. The fixed organoids were placed in gelatin, followed by routine tissue processing and embedding. Xenograft tissues was harvested from mice and fixed in 10% formalin and embedded into paraffin. Haematoxylin–eosin (H&E) staining was performed using standard protocols on 4 mm paraffin sections. Immunofluorescent (IF) multiplex method is described in S2 Fig.

More than 400 PDXO and PDX models were evaluated by pathologists for similarities morphological characteristics such as cells with large irregular nuclei, large basophilic nucleoli and frequency or abnormality of mitoses, formation of glandular structure in the adenocarcinoma models and squamous epithelial differentiation in the squamous cell carcinoma models.

### Genomic characterization

Organoids were collected from the culture well, followed by centrifugation at (12000g for 5 minutes (4°C). The pellet was collected by removing the medium supernatant and snap frozen (dry ice) in a microtube and then transferred to -80°C freezer. RNA or DNA were extracted using standard procedures, and NGS analysis for both RNAseq and whole exome sequencing (WES) were performed as previously described [9, 36]. PDXO authentication was verified by a 200-SNP panel using deep NGS sequencing. PCA is described in S3 Fig.

### Drug screening 384 well plate format

The method for screening organoids has been previously described [41]. Briefly, organoids were suspended in 2% (v/v) Matrigel in 384-well plate, with a seeding density of 200–1000 PDXOs per well and incubated at 37°C for 6–12 hours. Test agents were added to each well in triplicate according to a drug dilution scheme for 9 doses, in serial dilution by digital dispenser, including a negative control vehicle (with 100% viability) and a postive control of 5 μM starurosporine (0% viability or 100% kill). The plates were incubated at 37°C for 5 days [36] and organoid cell viability was determined using CellTiter-Glo^®^ as per the manufacturers instructions on a luminescence multi-well platereader. The normalized viabilities of each well were calculated, and dose-response curve and absolute IC_50_ values were created by non-linear curve fitting. An *in vitro* IC_50_<1μM and a dose response with almost 100% kill was referred to as sensitive whereas an IC_50_>1μM and/or 100% kill not achieved was not sensitive. Partially sensitive was referred to when the IC_50_<1μM but 100% kill not achieved or IC_50_>1μM and 100% kill achieved.

### PDX *in vivo* treatment

The evaluation of anti-tumor activity using PDXs has been described previously [7, 8, 10]. When TV reached on average 100–200 mm^3^, mice were grouped equally by tumor volume into treatment and vehicle control groups, each group comprising 5–10 mice and treatment initiated. Tumor growth inhibition (TGI) was calculated as TGI % = 1 - ΔT/ΔC where T and C are the mean tumor volume of the treated and control groups, respectively. An *in vivo* TGI>70% was referred to as sensitive to treatment, whereas TGI<40% was not and TGI 40–70% was referred to as partial responsive with progressive disease.

### Generation of engineered PDXO

To generate the engineered LI6664-Luc, LI6677-Luc, and LI6677-CD19-Luc PDXOs, specific constructs (firefly luciferase, human CD19, and combination of CD19 and Luc) were designed using the lentiviral expression vector pLVX-EF1a-Puro. Expression constructs were validated by transient transfection followed by western blot. Lentivirus was then packaged using the third generation of packaging plasmids which were transfected together with the lentiviral expression vector into 293T cells. Packaged lentivirus was titrated and used to transduce the target PDXO organoids. Organoids were split 2–3 days before transduction and then collected and dissociated into single cells on the day of transduction. Then lentivirus supernatant was applied to 0.5 x 10^6 organoid single cells in 1 mL growth medium with 10 μM Y-27632 (Abmole Bioscience Inc), a Rho-associated, coiled-coil containing protein kinase (ROCK) inhibitor, and incubated overnight. The next day, cells were harvested and washed twice before plating in 70% Matrigel. Cells were grown in culture radium with 10 μM Y-27632. Transduction of PDXO models was confirmed either by western blot (hCD19), or luciferase activity assay (Luc). For LI6677-CD19-Luc organoids, CD19 beads were used to purify the mixed pools. Engineered models were SNP confirmed and banked for future assays.

### Establishment of organoid-derived xenograft

Approximately 2x10^3^ organoids were mixed with Matrigel (1:1 ratio) and injected subcutaneously in immunocompromised mice and tumor dimensions measured twice weekly, as described above. For orthotopic implantation of bioluminescent organoids, fragments of approximately 2-3mm diameter were inoculated into the liver. Mice were imaged under anesthesia using the IVIS^®^100 imaging system (Caliper Life Sciences) 15 minutes after administration of a luciferase substrate, D-luciferin (intraperitoneal, 60 mg kg^−1^ in sterile PBS). Areas of luminescence were identified as Regions of interest (ROIs) and quantified as photons emitted using Living Image/Igor Pro Software (Caliper Life Sciences).

### Tumor organoid ADCC assay

The expression of tumor associated antigens (EpCAM and HER2) in PDX was measured by RNAseq. To confirm the expression by the corresponding PDXOs, tumor organoids were dissociated to single cells using TrypLE Express, and stained with corresponding antibodies and measured by flow cytometry. ADCC on HER2^+^ tumor organoids was evaluated by co-culture of organoids with PBMC at a ratio of 50:1 and incubation with anti-Her-2 antibody for 4 hours. Lactate dehydrogenase (LDH) release assay was performed to measure tumor organoid killing using CytoTox 96^®^ Non-Radioactive Cytotoxicity Assay kit (Promega).

### CAR-T mediated tumor organoid killing

Organoids were expanded and digested into single cells with TrypLE (Thermo Fisher, CAT#12605036). The expression of hCD19 and hEpCAM were measured by flow cytometry. Confluent organoids were collected, sheared and plated one day before the coculture with CAR-T cells (CAR-T-hCD19, NeoLogic Bio, LtdD; CAR-T EpCAM, Yihao Bio, Ltd.). On the day of the co-culture, organoids were harvested, filtered with 70- and then negatively filtered with 20- μm-strainers, counted and 1x10^5^ organoids digested by TrypLE into single cells to obtain an estimation for the number of single cells per organoid. Organoids, within 20~70 μm, were seeded in 96 ULA U-Bottom plates at a density of 1000 organoids/well in 5% Matrigel. The CAR-T cells were thawed and incubated with the organoids with specific effector:target (E:T) ratios (CAR-T cells:organoid single cells). Luciferase activity was measured after 48-hour incubation with Steady-Glow Luciferase kit (Promega CAT#E2510) using a PerkinElmer multilabel reader. Concentration of IFN-γ or Granzyme B was measured after 24- or 48-hours incubation using ELISA kits (R&D, CAT#DIF50, CAT#DGZB00).

### Statistical/data analysis

A sigmoidal dose-response curve was fitted using a nonlinear regression model to calculate IC_50_ values. Absolute IC_50_ is calculated where y-axis was set at 50% using the GraphPad Prism 8.3.1 software. Correlation and linear regression analysis of IC_50_ values was performed using log10 transformed values with Pearson’s correlation. Students t-tests and linear regression were applied for comparing the data of the two groups (SPSS version 19.0). P<0.05 was considered statistically significant. Intra-plate variation was assessed via Z’ factor as previously described [42].

## Results

### 3D organoid growth of PDX origin and creation of a matched PDX:PDXO biobank

**Fig 1:**
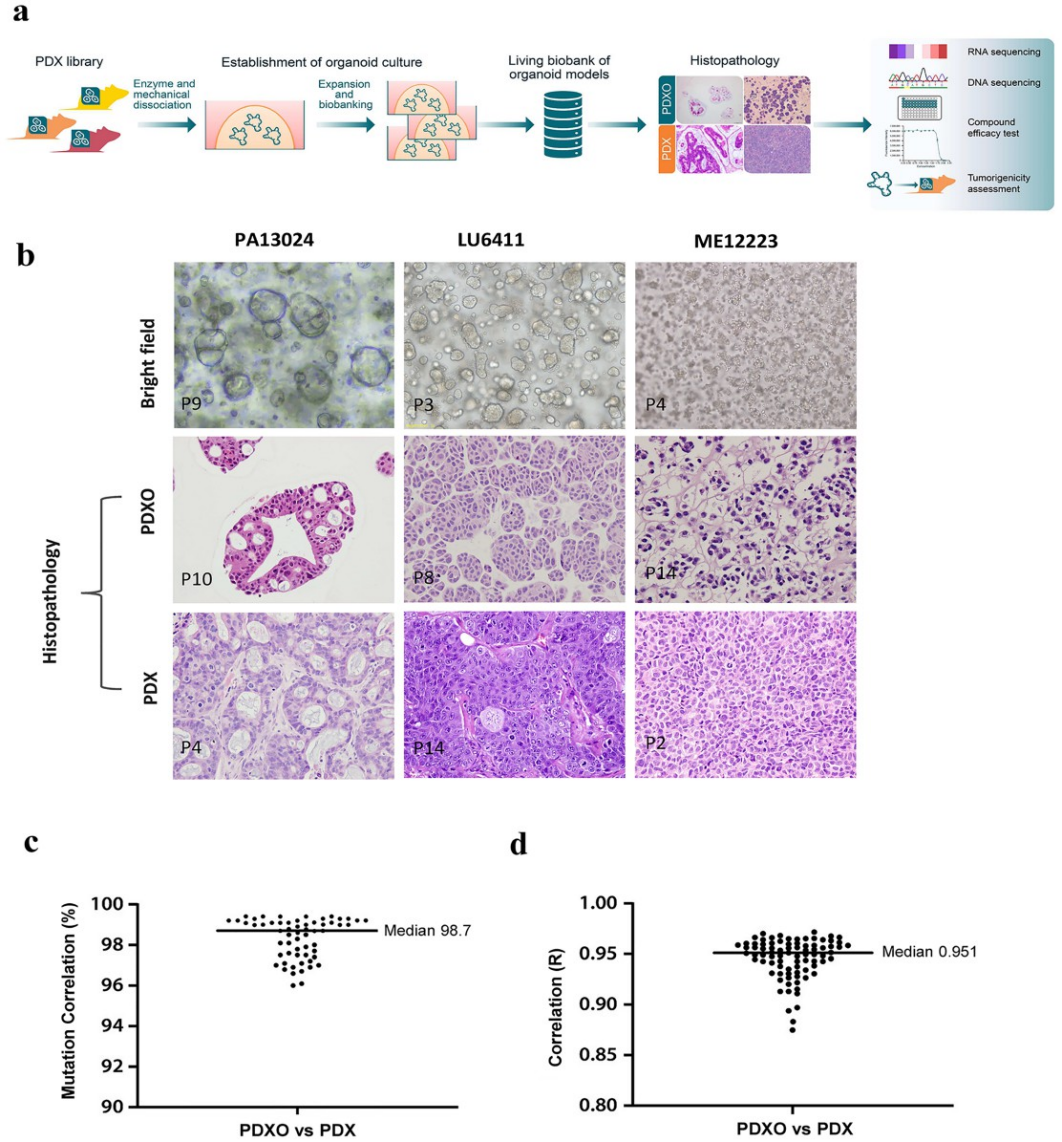
Establishment and characterization of a PDXO biobank. a) Schematic workflow of the establishment of PDXOs from PDX library of Western and Asian patient origins and characterization of banked models. b) Bright field (top panel) and H&E staining (histopathology, middle panel) of three selected PDXO models (pancreatic tumor, lung tumor and melanoma), showing cystic, compact and grape-like phenotypes, and H&E staining of matching PDX (bottom panel); c) WES analysis of 59 matched PDX/PDXO data sets from 9 different cancer types (colon, lung, gastric, pancreatic, ovarian, breast, cholangiocarcinoma, liver and bladder) and d) RNAseq expression analysis from 83 matched PDX/PDXO data sets across 11 cancer types (colon, lung, gastric, pancreatic, ovarian, breast, cholangiocarcinoma, liver, bladder, esophageal and melanoma) with median values shown.

We set out to build a PDXO biobank by converting our existing PDX library [5], using methodology previously described to create organoids from adult stem cells [18, 21, 36, 43]. The basic workflow for establishing the PDXO biobank is depicted in Fig 1a, where PDX tumors were freshly harvested from mice, or from cryopreserved PDX tumor fragments, minced, cells isolated by enzymatic digestion and mechanical disruption and then seeded into Matrigel domes to create an organoid culture which is expanded and banked once growth is established [36]. We created a large panel of ~550 PDXOs from 17 different types of carcinomas (S1 Table). The established organoids generally had varying growth kinetics, with culturing time ranging predominantly from 7–14 days before requiring passaging, similar to those described for PDOs by others. In addition, PDXOs could be cryopreserved with nearly 100% recovery rate, which was critical for establishing a living biobank as well as the PDXO identity authenticated by single-nucleotide polymorphism (SNP) as previously reported [32]. Using bright-field microscopy, the morphology of PDXOs look similar to those typically observed for PDOs [23, 34, 43], inclusive of cystic, compact [36], grape-like and budding morphologies (representative morphology phenotypes are shown in S1a Fig, with selected models shown in Fig 1b), supporting the hypothesis that PDXO structure and morphology is generally similar to those seen for PDOs. Following establishment of organoid cultures and the associated biobank we next set out to characterize the organoids in comparison to their originating PDX.

### PDXOs share similar histo- and molecular pathology as their parental PDXs

PDXs and PDOs independently have been reported to share similar histo-/molecular pathology to the original patient tumors [3, 5, 19, 23, 24, 28, 44]. Histopathology analysis of H&E stained PDXO sections were compared to H&E staining of parental PDX tissue, which indicated that each PDXO, despite only retaining the epithelial component, preserved the histopathology of the parental PDX (Fig 1b and S1b Fig) across the cancer types. The presence of CSCs were also confirmed in PDX and PDXO colorectal cancer (CRC) model pairs for the expression of common stem cell markers (*e*.*g*. Lgr5 [18], CD44, CD133, SOX2), tumor cell surface marker EpCAM and pan-CK, a basal cell marker [45] (S2 Fig).

Next, we set out to profile PDXOs for their genomics, including transcriptome sequencing (RNAseq), as already reported for the original PDX models [5], and whole exome sequencing (WES) [36] in order to generate a well-annotated PDXO collection. Genomic DNA from PDXOs and corresponding PDXs were analyzed by WES for changes at the DNA level, including single nucleotide variants (SNVs). Across a panel of 59 matching pairs of PDXO/PDX data sets from 9 different cancer types, high mutational concordance (median mutation correlation 98.7%) was observed, demonstrating genetically near-identical between the pairs (Fig 1c, individual representative data shown in S2 Table). Similarly, the gene expression of the transcriptome for 83 corresponding models across 11 cancer types also demonstrated high correlation as shown in Fig 1d (median R value 0.951) and S2 Table, with PCA analysis for CRC shown in S3a Fig. In addition, the mutational concordance and gene expression levels across passages, ranging from a passage difference of 1 to 15, remained relatively stable (S3b & S3c Fig). These observations confirm that PDXOs largely preserve the genomic and transcriptomic features of the originating tumors.

### PDXO 384-well format *in vitro* cytotoxicity assay

**Fig 2:**
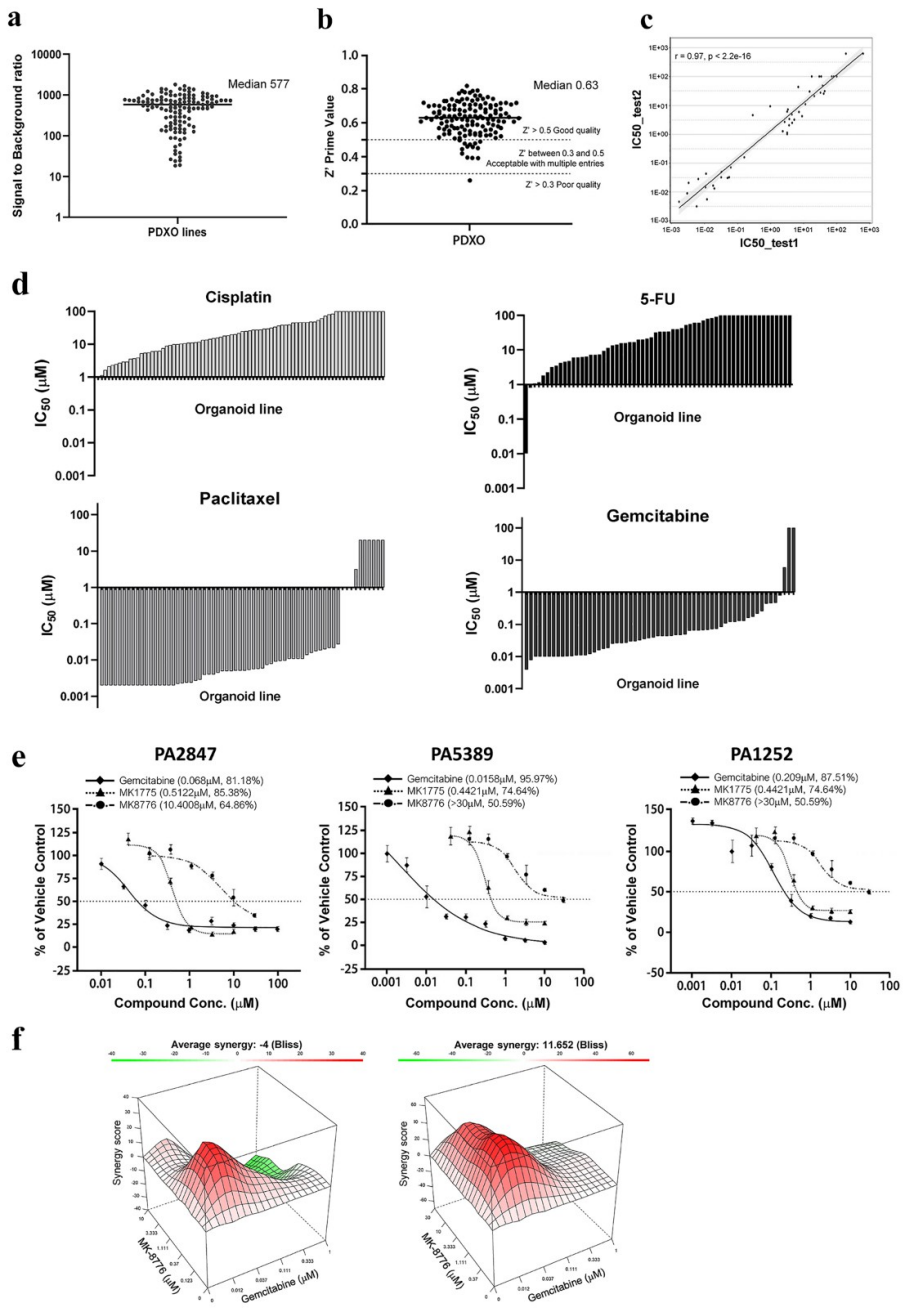
PDXO 384-well plate assay is highly reliable, robust and suitable for HTS. a) Signal to noise ratio across 129 PDXO lines as defined by the luminescence ratio of vehicle/positive control with median value shown; b) Z-prime for intraplate analysis of 138 PDXO models in a 384-well format with a median value above industry standards; c) Interplate analysis (52 pairs of IC_50_ data sets across 2 passages shown on each axis for 6 models) with r value of 0.97 and r^2^ value of 0.94 (Pearson’s correlation). d) SOC (cisplatin, paclitaxel, gemcitabine and 5-FU) response across multiple models from different cancer types with representative IC_50_ values plotted in waterfall format, where IC_50_<1μM was classed as sensitive; e) Pancreatic PDXOs (PA2847, PA5389 and PA1252) dose-response (% of vehicle control ± SEM) to gemcitabine (♦), MK1775 (▲) and MK8776 (●); f) 3D response surface plot of synergy score via Bliss mathematical model for gemcitabine + MK1775 (left) or gemcitabine + MK1775 MK8776 (right) in the PA2847 PDXO model with red representing high scores and green representing low scores.

We next examined the reliability of our PDXO models in a 384-well assay format using CellTiter-Glo^®^ (CTG) as an endpoint readout of cell viability [8, 36, 41]. Our results demonstrated that the quality of PDXO screening was high, with a median signal to noise ratio of 577 across 129 PDXO models (Fig 2a). Furthermore, the z’ factor for the PDXO screen, reflective of intraplate precision [42], reached relative high values with a median of 0.63 (Fig 2b). Both parameters are significantly above the acceptable robust HTS standards. In addition, we have also tested interplate variability by comparing the IC_50_ values generated for a range of compounds across several models over 2 passages (Fig 2c). The correlation of IC_50_ values across experiments for each agent was analyzed by linear regression and generated a R^2^ value of 0.94 (p<0.0001, Pearson’s correlation), similar to that of cell lines (R^2^ value 0.96, p<0.0001 S3a Fig).

We have also established 274 PDXs as *ex vivo* cultures (S1 Table) for *in vitro* assays (see S4 Fig). However, the *ex vivo* cultures could not be maintained in continuous culture and only cryopreserved and reanimated into cytotoxic assay. In addition, the R^2^ value of 0.63 was observed when the correlation of IC_50_ values across experiments was assessed (S4a Fig), which was significantly lower than that observed for PDXOs (Fig 2c) and cell lines (S4b Fig), thus demonstrating high interplate variation. Taken together, the PDXO 3D screen demonstrated to be a robust assay similar to other *in vitro* assays, but with greater reliability and reproducibility over 3D *ex vivo* PDX assays.

### Rapid identification of drug-sensitivity and optimal combination strategies

With the establishment of robust HTS workflow, we could rapidly screen large panels of PDXOs across cancer types with commonly used standard of care (SOC) chemotherapies (Fig 2d). The majority of models showed an insensitive phenotype to cisplatin and 5-FU, whereas a large proportion of models were relatively more sensitive to paclitaxel and gemcitabine, which could be used to select models for *in vivo* testing or combination evaluation. Since testing combinations *in vivo* is challenging, *in vitro* assays have intrinsic advantages in characterizing drug-drug pharmacological coordination, such as synergistic, additive or antagonistic effects, as compared to the *in vivo* setting as multiple numbers of drugs and potential combinations can be tested if HTS is used, including a matrix of different drug concentrations for each drug and without the complexity of pharmacokinetic/drug metabolism. To this end, we have used a 384-well HTS format to assess the synergistic effects of different drugs in PDXOs.

Synergies between MK-1775 (WEE1 inhibitor) and MK-8776 (CHK inhibitor), and each with gemcitabine have been observed in tumor cells, including melanoma, colon cancer and p53 mutant pancreatic cancers [46–50]. To verify these synergies in PDXOs, we selected p53 mutated pancreatic PDXOs, PA5389 and PA1252 with a pR282W and pR273H p53 mutation, respectively, and PA2847 with a nonsense mutation (pW91Ter) and examined the response to gemcitabine, MK1775 and MK8876 (Fig 2e). All models were sensitive to gemcitabine and MK1775 with IC_50_ values <0.2μM and <1μM, respectively, whereas MK8776 generated IC_50_ values of >10μM. Following this, each PDXO was treated with a 6-point titration of each inhibitor and combined in a 6x6 matrix format for 5 days and cell viability measured. Two independent mathematical models of synergy, Bliss and Loewe [51–53], were used to assess the combination effect, with synergy score >5 indicating synergy. The combination of MK-1775 and MK-8776 shows very strong synergistic effect in all of the organoid models tested (S5a Fig). The average synergy score was greater than 10 for 2 of the PDXOs (Table 1) using either of the synergy models with the highest score greater than 50, suggesting very strong synergistic interaction between the two drugs.

**Table 1:** Summary of synergy of combination drug treatments in pancreatic PDXOs.

Drug combination	PDXO	Highest Synergy Score		Average Synergy Score	
		Bliss	Loewe	Bliss	Loewe
**MK1775 + MK8776**	**PA5389**	50.7	71.6	12.822	13.619
	**PA2847**	52.92	51.71	10.556	10.594
	**PA1252**	54.81	8.82	38.62	-5.29
**MK1775 + gemcitabine**	**PA5389**	48.8	44.57	8.402	14.947
	**PA2847**	32.19	15.34	-4	-5.043
	**PA1252**	58.72	44.75	14.234	12.065
**MK8776 + gemcitabine**	**PA5389**	64.08	56.78	19.925	21.492
	**PA2847**	60.1	42.64	11.552	-4.805
	**PA1252**	79.31	32.273	70.89	27.614

In combination with gemcitabine, both MK-1775 and MK-8776 shows very strong synergistic effect in all the organoid models (S5b and S5c Fig) with average synergy score was greater than 10 in the p53 mutated models consistent with previous reports [46, 47]. The highest synergy scores >30 were seen with MK8776 and gemcitabine for both mathematical models (Table 1). However, some low average values were also observed suggesting antagonism with certain dose combinations. The highest synergy scores across similar concentrations for gemcitabine/MK1775 and gemcitabine/MK8776 were compared for each PDXO (S5d Fig). The peak of the synergy score was significantly greater for gemcitabine/MK8776 than gemcitabine/MK1775 in PA2847-PDXO (containing a nonsense p53 mutation) identified by the Bliss mathematical model (Fig 2f), which is more compatible for non-interacting drugs that elicit responses independently (*e*.*g*. by targeting separate but complement pathways). Overall, the rapid screening of relevant organoids in a combination matrix approach enabled synergistic profiling to be easily conducted providing valuable insight into combination strategies.

### *In vitro* PDXO pharmacology correlates with *in vivo* PDX pharmacology

**Fig 3:**
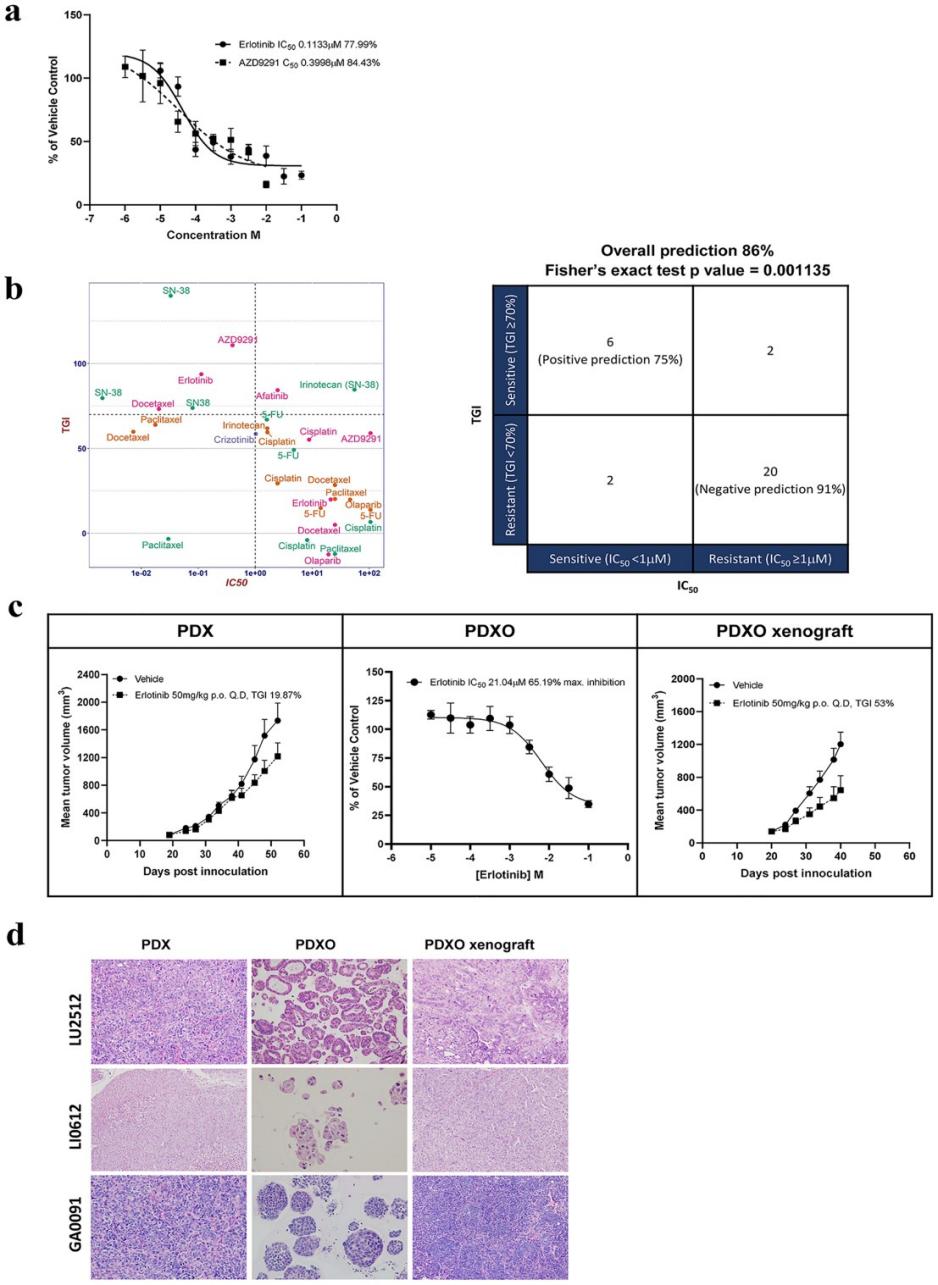
High correlation between *in vitro* PDXO and *in vivo* PDX pharmacology. a) NSCLC LU1235 (EGFR activation exon-19 deletion) PDXO dose response (% of vehicle control ± SEM) to erlotinib and AZD9291 to determine absolute IC_50_ values and maximum inhibition; b) Matched PDXO *in vitro* and PDX *in vivo* responses across 30 PDXO/PDX paired datasets (left) to determine the predictive power of PDXO for PDX response. The criterion for *in vitro* and *in vivo* sensitive/responsive or resistant/non-responsive is shown on the right panel with number of datapoints in each category to determine the predictive values and correlation analysis by Fisher’s exact test (right). c) Growth and therapeutic response to erlotinib in NSCLC LU2512 (EGFR wildtype) PDX *in vivo* (mean tumor volume ± SEM with 5mg/kg IP weekly dose regimen for erlotinib, left), PDXO *in vitro* (middle, dose response curve of % of vehicle control ± SEM to determine absolute IC_50_ values and maximum inhibition) and PDXO xenograft *in vivo* (TGI with 5mg/kg IP weekly dose, right). d) H&E staining of three models (top to bottom; lung cancer LU2512, liver cancer LI0612 and gastric cancer GA0019) grown as either PDX (*in vivo*, 40x magnification, left), PDXO (*in vitro*, 16x magnification, middle) or PDXO (*in vivo*, 40x magnification, right).

PDXs have been shown to be particularly useful in assessing targeted therapies on subjects with specific oncogenic driver mutations [7, 8, 10, 54]. To test targeted treatments, we selected PDXOs with similar relevant driver mutations. PDXO-LU1235 which harbors a common EGFR activation exon-19 deletion [7, 8] was treated with the first-generation EGFR inhibitor, erlotinib and a third-generation inhibitor AZD9291 at various concentrations in 384-well plate to establish an IC_50_ value. PDXO-LU1235 showed sensitivity to both erlotinib and AZD9291 (IC_50_ < 1μM, Fig 3a & Table 2), which was consistent with the complete tumor regression that was observed in PDX *in vivo* testing for erlotinib [7] and AZD9291 (unpublished data). In comparison, PDXO-LU2512, which does not carry an EGFR activation mutation, showed poor response to erlotinib (IC_50_ >20μM, Fig 3c) similar to its parental PDX model (TGI<20%). In addition, SOC cisplatin induced an IC_50_ of 8.8μm in PDXO-LU2512 and 4.9μM in LU1235 which translated to an insensitive/partially sensitive response in the corresponding PDXs (TGI 55% and 60%, respectively).

**Table 2:** Correlation of NSCLC LU1235 PDXO and PDX pharmacology.

PDXO *in vitro* LU12350				PDX LU1235 [7]		
Drug	IC_50_ (μM)	Max Inhibition (%)	Drug Sensitivity	Treatment Regimen	Median TGI (%)	Drug Sensitivity
Erlotinib	0.1133	77.99	Sensitive	50mg/kg, p.o., q.d.	107	Sensitive
AZD9291	0.3998	84.43	Sensitive	10mg/kg p.o. q.d	110	Sensitive

We next investigated the pharmacological correlation of PDXO *in vitro* response with *in vivo* PDX response in a large panel of paired PDXO and PDX models in order to assess the overall potential of PDXO to predict *in vivo* outcome to drug treatment. To this end, we examined a panel of 5 SOC chemotherapies and 7 targeted agents, across 4 cancer types and 13 PDXO/PDX matched pairs where drug effects were categorized as either sensitive or insensitive based on specific criterion for the resulting IC_50_ value and TGI (Fig 3b). Statistical analysis of 30 data points indicated that *in vitro* and *in vivo* pharmacology characterizations were not independent, and that *in vitro* response was predictive of the *in vivo* outcome with overall ~86% accuracy (p = 0.001134, Fisher’s exact test, Fig 3b), with positive predictions of 75% and negative prediction of 91%. In comparison, PDXs in 3D *ex vivo* assay had a predictive power of 68%, with positive prediction of 27% and negative prediction of 92% when 40 different drug treatments were compared to the corresponding *in vivo* PDX response (S4c Fig). Our data suggest that PDXO *in vitro* pharmacology has overall good predictive power for the corresponding PDXs *in vivo* in comparison to 3D *ex vivo* PDX assays in addition to higher reproducibility (Fig 2c).

### PDXO are tumorigenic and similar to their ancestry PDX

PDXOs were inoculated into immune-compromised mice to test their tumorigenicity and tumor growth kinetics. We found tumor growth kinetics to be similar to those of the parental PDX (Fig 3c). The response to erlotinib in non-small cell lung cancer (NSCLC) LU2512 (EGFR wildtype) PDX *in vivo* (TGI 19.87% with 5mg/kg IP weekly dose), PDXO *in vitro* (IC_50_ value of 21.04μM and 65.19% maximum inhibition) and PDXO xenograft *in vivo* (53% TGI with 5mg/kg IP weekly dose) was comparable with poor sensitivity to erlotinib reflected in all three scenarios (Fig 3c). The H&E staining of PDXO, PDX, and PDXO-derived tumors across various cancer types demonstrated consistent histopathology (Fig 3d). This further supports the hypothesis that PDXOs are “biologically equivalent” to the parental PDXs.

### Engineering reporter-PDXO to enable imaging analysis

**Fig 4:**
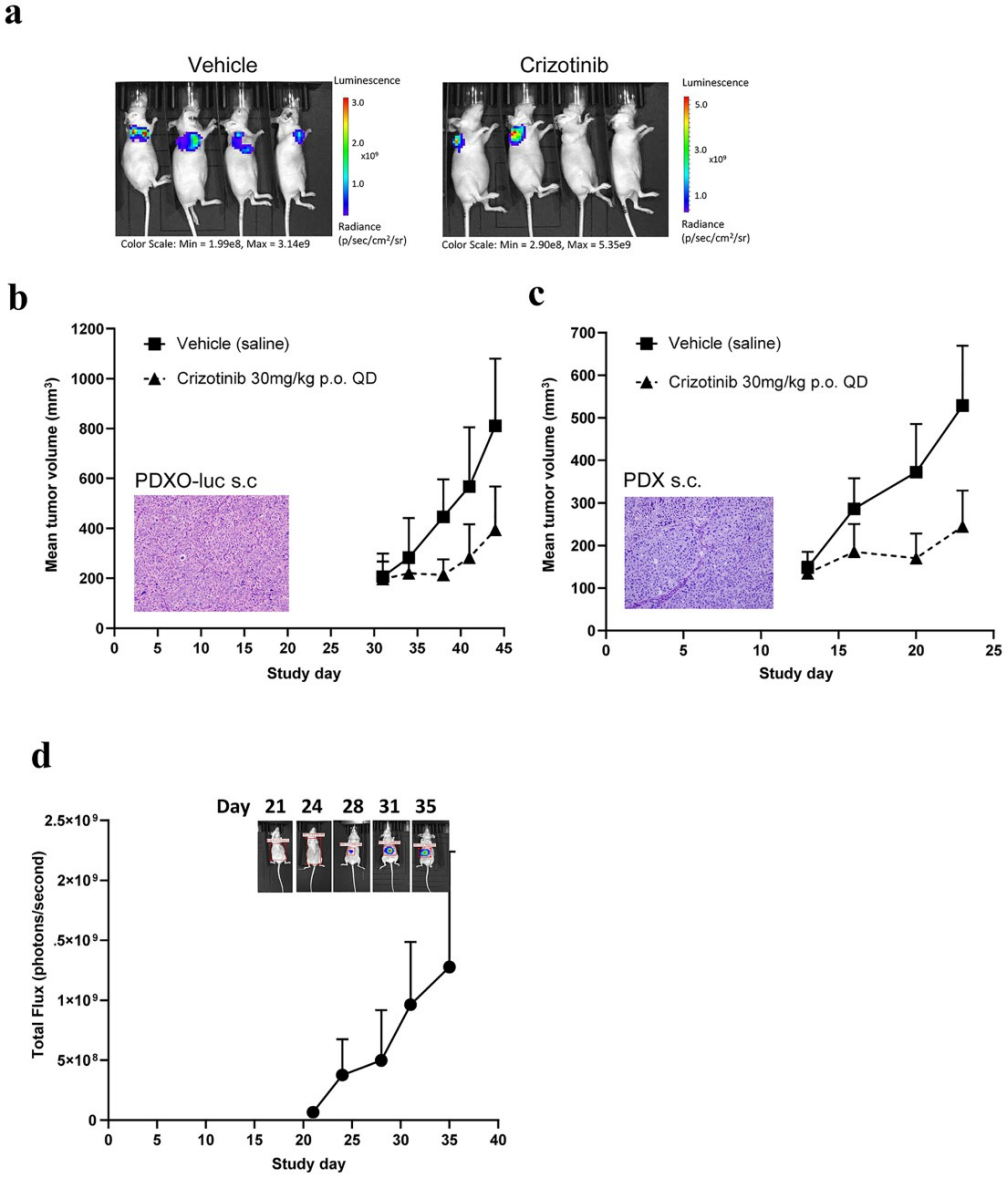
Organoid engineering enables luciferase tagging of PDXOs for real-time tracking. a) *In vivo* subcutaneous imaging of LI6664-luc PDXO to confirm tumorigenicity of engineered organoid and bioluminescent signal of construct; b) Subcutaneous LI6664-luc PDXO xenograft morphology (H&E, 20x magnification) and therapeutic response to crizotinib (mean tumor volume ± SEM, 30mg/kg p.o. daily crizotinib dose regimen) compared to original subcutaneous LI6664 PDX and c) Real-time optical imaging (mean total flux ± SEM) of orthotopic LI6664-luc PDXO implanted in the liver to determine growth kinetics in the liver with representative mouse image for each day of imaging (ventral view).

One of the application limitations of PDXs is the challenge associated with engineering the models, *e*.*g*. introducing luciferase reporters and/or overexpression of human tumor associated antigens (TAA), creating drug resistant mutations, *etc*., due to the limited ability to culture PDX *in vitro* or to engineer directly *in vivo*. We successfully engineered the stable *in vitro* PDXO cultures, for both *in vitro* and *in vivo* applications via lentiviral transduction. LI6664-PDXO was efficiently transduced with a luciferase gene under ubiquitous promoter via lentiviral transduction and implanted subcutaneously to firstly establish tumorigenicity and bioluminescent signal (Fig 4a). Similar to the parental PDX, LI6664-PDXO was *c-met* amplified (copy number variant determined by WES >20 for both PDX and PDXO), therefore the response to crizotinib was tested in the PDXO xenograft and compared back to the original PDX (Fig 4b & 4c respectively). Both the response to crizotinib (measured by tumor volume) and the histology (by H&E staining) were equivalent to the original PDX. Implantation of LI6664-luc PDXO into the liver was also successfully established with longitudinal growth measured by optical imaging enabling quantification of the liver tumor (Fig 4d). The efficient transduction of PDXOs, retention of biological features and quantification by imaging will facilitate the use of patient relevant tissue for both *in vivo* and *in vitro* applications as well as the generation of new models.

### Organoid co-culture systems to investigate immune-oncology modality

IO is an area of intensive research due to the successful development of new immuno-therapeutics and the rapid growth of knowledge on the role of tumor microenvironment (TME) interactions, particularly tumor-infiltrating leukocytes (TILs), including T-cells. The limitations of current IO animal modelling calls for alternative model systems [6]. Recapitulating the TME by co-culturing tumor organoids with immune cells becomes highly attractive, providing an efficient and defined approach to assess immune modulatory and tumor killing effects of investigational IO therapeutics, including monoclonal antibodies, CAR-T cells, CAR-NK and small molecules [55, 56].

Here, we co-cultured a PDXO (GA0091) with allogeneic peripheral blood monocyte cells (PBMCs) to assess the effects of allogeneic T-cell killing of PDXOs. CFSE-labeled GA0091-PDXO were co-cultured with activated (72hr-anti-CD3/CD28 antibodies) allogeneic PBMCs, where organoid killing by allogeneic T-cells was assayed by flow cytometry using a live/dead dye (S6a Fig). The results demonstrated allogeneic T-cell mediated organoid killing as shown in S6b Fig.

**Fig 5:**
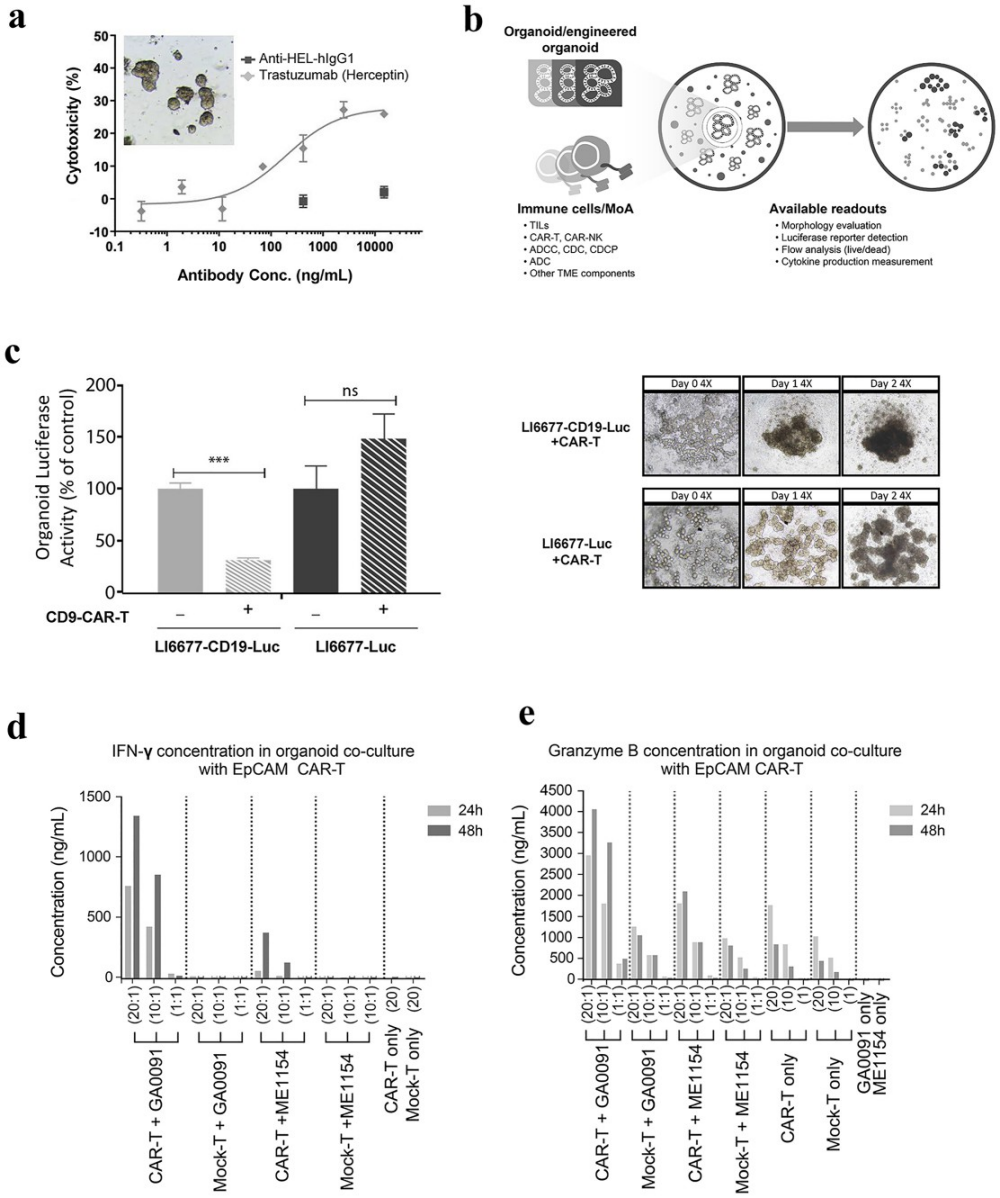
Co-culture of PDXOs with immune cells. a) ADCC-mediated killing of HER2^+^ ovarian tumor organoid OV0250 (inset bright field image of organoid culture) was determined via co-culture with PBMCs and Herceptin^™^ and quantified by flow cytometry (mean % cytotoxicity ± SEM); b) Schematic of co-culture systems with immune cells or mechanism of action (MoA) and different types of endpoint readout; c) CAR-T cell mediated specific killing of liver cancer organoid (PDXO-LI6677) engineered to express both human CD19 and luciferase (grey shaded bars) or just luciferase (black shaded bars) co-cultured with (hatched bars) and without (solid bars) CD19 CAR-T cells at 1:10 ratio for 2 days with luciferase activity used to track organoid growth/killing (% of control without CAR-T ± SEM). Representative bright field images (4x magnification) shown on right to show morphology changes in the coculture of LI6677-CD19-luc PDXOs (top images) or LI6677-luc PDXOs (bottom images) at different timepoints. d) Co-culture of EpCAM^+^ gastric cancer organoid (PDXO-GA0091) with EpCAM-CAR-T cells led to CAR-T cell activation/mediated killing, compared to the EpCAM^-^ melanoma organoid (PDXO ME1154) as measured by d) IFNγ and e) Granzyme B ELISA over 24 (grey bars) and 48 (black bars) hours.

Antibody dependent cellular cytotoxicity (ADCC) is one of the important MOA of many monoclonal antibody cancer therapies, including Herceptin^™^ (against HER2) and Erbitux^™^ (against EGFR). We next tested the ADCC by Herceptin^™^ against a HER2^+^ ovarian PDXO, PDXO-OV0250, which was confirmed to express surface HER2 from a panel of PDXOs (S6c Fig). In a co-culture of both PDXO-OV0250 and PBMC in the presence or absence of Herceptin, similar to a standard ADCC assay, we demonstrated effective specific killing of PDXO-OV0250 through ADCC MOA as monitored by flow cytometry (Fig 5a). Thus, PDXO co-culture could also be a good candidate system to evaluate antibody drug targeting tumor associated antigens.

Next, we tested the killing of PDXO by CAR-T cells in a co-culture system with different pairs of CAR-T cells and PDXOs using different endpoint readout methods (Fig 5b). First, we engineered a luciferase expressing liver tumor PDXO, PDXO-LI6677-luc to over-express CD19 (S6d Fig). We then co-cultured PDXO-LI6677-CD19-luc with CD19-CAR-T cells and quantified the luciferase activity following co-culture. A significant reduction of luciferase activity was observed in the CD19^+^ PDXO co-culture only, suggesting the specific killing of CD19^+^ PDXO mediated by the CD19-CAR-T cells, as shown in Fig 5c (p<0.001, unpaired t-test).

In the second experiment, we tested EpCAM-CAR-T cells co-cultured with EpCAM^+^ gastric PDXO-GA0091 and EpCAM^-^ melanoma PDXO-ME1154, and measured the interferon-gamma and Granzyme B levels in the culture. Our result demonstrated CAR-T cell-specific activity reflected by the elevation of both interferon-gamma (Fig 5d) and Granzyme B (Fig 5e) in EpCAM^+^ gastric-PDXO-GA0091 co-culture as compared to the EpCAM^-^ melanoma PDXO-ME1154 co-culture. Collectively these results demonstrate the utility of an organoid co-culture systems for CAR-T efficacy and specificity as proof of concept (POC).

## Discussion

Thousands of PDXs have been established globally and enrolled as surrogate subjects in population-based pharmacology trials (“mouse clinical trial” format)and more recently, *in vitro* PDOs have been reported to mimic clinic patient responses (‘Patient In Lab^™^’) [18, 23, 24, 28, 43]. Here we demonstrate the application of similar methods used to create PDOs from patient tumor tissues to create a biobank of PDXOs from our library of PDXs, providing a unique collection of matched *in vitro* and *in vivo* preclinical patient-derived models. Through genomic, histopathological examination and pharmacological profiling of this paired PDX/PDXO-library, we confirmed “biological equivalency” between PDX and the corresponding PDXO, including the presence of CSC markers. This also supported the notion that both PDX/PDXO represent the same disease characteristics derived from the same ancestral CSC (the original CSC of patient tumors). With this “equivalency” or “predictive power” in mind, we report several advantages of organoids, as compared to xenografts, in enabling adoption for HTS within pharmaceutical development: 1) *in vitro* screening of large compound libraries to identify leads; 2) screening of combination treatments, allowing identification of new or complex combinations; and 3) screening of large libraries of patient derived disease models to identify indications or genetic targets for target discovery. Where PDX have generally failed or proven ineffective, PDXO biobanks, in this case, are of particular value since it now enables us to take advantage of the already available large patient-derived libraries. Although 3D *ex vivo* assays have taken advantage of the existing large collection of highly-predictive PDXs by enabling pharmacology testing in an *in vitro* setting to inform on *in vivo* testing [57, 58], they have not been widely adopted in the industry due to the reliance on PDX material, combined with high unreliability and variability making data interpretation difficult and unsuitable for HTS.

The engineer-ability of PDXO enables efficient gene editing of patient-derived models, which otherwise has been difficult to achieve with PDXs, rendering broad research applications possible, *e*.*g*. *in vivo* orthotropic imaging, introduction of resistance, TAA expression, target validation, *etc*. A number of methods have proven useful in delivering gene editing into organoids, electroporation, lentiviral transduction and CRISPR [59–61]. Furthermore, genome-wide screens using RNAi- or sgRNA libraries [62] could be powerful tools for discovery of new drug targets.

Co-culture of PDXOs with different components of the TME enables a reductionist approach to investigating the roles of different interactions between tumors and the TME, as reported by others [55, 56] and in this report. It can readily be employed for POC study of new IO treatments and for examining the modulation of an existing IO therapy through combination. Co-culture systems can also be readily used for large-scale IO screening for models or candidates, such as biologics and small molecules. Furthermore, co-culture avoids the undesirable graft-versus-host-disease (GvHD) occurring in humanized mouse experiments [6]. All these unique features of PDXOs make them a powerful model system for IO, superior to PDX.

*In vivo* PDX and *in vitro* PDXO models have different pros and cons, and therefore certainly have different utilities in pharmacology and translational research, which can also complement each other. For example, unlike PDX tumors PDXOs lacks TME and ability to assess pharmacokinetics and toxicology of drug effects, thus *in vivo* assays are still absolutely essential. It is also worth noting that there may be some differences between PDXO and PDO. First, while PDO from less transformed tumor tissues can be established, PDXO always represent more malignant tumors since less transformed tumors may not grow in mice [44]. However, it must be noted that the establishment success of PDXOs from PDXs is lower than that observed for PDOs directly from the patient, which may be due to lack of human stroma to support the initial growth in 3D. Second, for PDXO, no matched normal control can be created as normal tissue does not establish in a xenograft model whereas PDO can be established from both normal and tumor tissue from a patient. In addition, one may need to pay attention to remove certain mouse components contaminating PDXO culture to improve successful establishment of organoid cultures. Nonetheless, converting the existing library of annotated PDXs as reported here provided a fast and productive approach to building a practical organoid biobank, thus creating a powerful translational tool for the early stages of drug screening as well as matrix-screens for both models and candidate simultaneously, which can then be readily validated *in vivo* using matched PDX models.

It is important to emphasize that accurate pharmacological predictions among different platforms (PDX trial/organoid screen), as well as in clinics, is largely dependent on relevant dose ranges (*e*.*g*. clinic relevant dose range) for each individual drug, instead of using arbitrary dose range calls for efficaciousness or responsiveness. Once the drug-specific clinically relevant dose range is determined, organoids can be readily deployed as a powerful translational tool, accelerating new cancer medicine advancement with reduced attritions and guide precision treatments.

## Supporting information

S1 Table Models established across different cancer type from PDXs models as either PDXO cultures *in vitro* or for 3D *ex vivo* assays. For PDXO collections, the % success rate per cancer type was determined based on the number of models successfully biobanked versus the number of models that failed organoid establishment. In comparison the dissociated PDX in 3D *ex vivo* conditions could not be maintained in continuous culture nor cryopreserved and resuscitated, therefore hindering the generation of a living biobank. PDX *ex vivo* cultures were approximately 25–35% successful.
(TIF)

S2 Table Summary of mutation concordance and mRNA expression correlation across a panel of PDXO/PDX matched models. (TIF)

S1 Fig Pathology analysis confirms consistency between PDXO and associated PDX, preserving original tumor features across different cancer types. a) Typical cystic, compact, budding and grape-like organoid phenotypes were observed using bright-field microscopy (16x magnification); b) Bright field (top panel) and H&E staining (middle panel, 40x magnification) of PDXO models from different cancer types (colorectal, gastric, lung, pancreatic, liver and ovarian), and H&E staining of matching PDX (bottom panel).
(TIF)

S2 Fig Identification of cancer stem cell markers across PDX and PDXO. CSC analysis was performed on four CRC-PDX/PDXO pairs (CR1520, CR2258, CR5048, CR11372). Tyramide signal amplification (TSA)-based fluorescent multiplex IHC staining in the Leica Bond Rx automatic platform was performed using a 4-plex/5-color CSC panel: DAPI (Sigma, #9542), CD133 (Cell Signaling, #86781), CD44 (Abcam, ab51037), SOX2 (Cell Signaling, #14962), Lgr5 (Beyotime, #AF1582), EpCAM (Cell Signalling Technology, #14452) and basal cell marker panCK (Abcam, ab270305), followed by whole slide scan using the Vectra^®^ Polaris^™^ automated quantitative pathology imaging system (PerkinElmer) and quantified by HALO^™^ image analysis software (Indicalabs) and represented as % of positive cells in the total tumor cell count. Pan-CK and CD44 showed universal co-localization predominantly on the cell surface with broader patterns in corresponding matched models. CD133 displayed a predominantly intraglandular-like staining pattern in PDXOs. Lgr5 expression was high in PDXOs (~100% tumors positive) than PDX, except for CR11372, suggesting more stemness in PDXO. In contrast, SOX2 was broadly deficient in all CRC PDXOs and PDXs. EpCAM showed broader expression in PDXO and consistent with corresponding matched PDX models, which displayed same epithelial-derived pattern in both.
(TIF)

S3 Fig Genomic characterization of matched PDX and PDXO pairs. a. Mutation concordance of PDXO across late and early passage analysed by WES; b. Expression correlation analysed by RNA seq. c. For principal component analysis (PCA) of colorectal models we first filtered out low expression genes, then we performed PCA based on the remaining genes. Prior to PCA analysis, gene expression data was normalized by variance stabilization transformations using DESeq2. Finally, we plotted the samples scores of first principle component (PC1) against the sample scores of the second principle component (PC2), while samples derived from PDX/PDXO were grouped into separate eclipses.
(TIF)

S4 Fig *Ex vivo/*i*n vitro* assay comparison. An *ex vivo* cell bank using cryopreserved *ex vivo* tumor cells dissociated from freshly isolated PDX tumor tissues. In brief, freshly isolated PDX tumor tissues were digested into single cell suspension using collagenase B. After washing and cell counting, the cells were cryopreserved in liquid nitrogen in standard freezing medium (complete medium supplied with 10% DMSO). Upon compound testing, cryopreserved PDX cells were revived and resuspended in 0.65% methylcellulose (final concentration) and loaded onto 96-well plates and cultured overnight in a 37ºC incubator with a supply of 5% CO_2_. Test compounds were then added in a 9-point dilution manner in triplicates. Following a 7-day incubation, cell viability was measured by CellTiter-Glo^®^ and plotted to determine IC_50_ values. Interplate analysis across a) 151 IC_50_ value datasets from different plates for 2D cell lines or b) 96 IC_50_ value datasets for PDX *ex vivo* 3D cultures analyzed for Pearson’s correlation. c) 3D *ex vivo* assay and *in vivo* efficacy correlation for PDX models across 40 datasets and 15 different models to determine the predictive power of 3D *ex vivo* assay system. The criteria for *in vitro* and *in vivo* responsive or non-responsive is shown on the right panel with number of datapoints in each category to determine the predictive values and correlation analysis by Fisher’s exact test.
(TIF)

S5 Fig Synergy evaluation in PA5389 and PA2847 pancreatic cancer PDXOs. Treatment for 72 hours with a) MK-1775 and MK-8776 combination; b) MK-1775 combination with gemcitabine and c) MK-8776 with gemcitabine represented as % inhibition or organoid death shown as a heat map for each concentration combination in matrix layout (left panel, red represents high, white represents low inhibition) and synergy score calculated using two mathematical models, Bliss (middle) and Loewe (right) and presented as a heat map where red represents synergy and green represents antagonism. d) Comparison of the synergy score for MK-1775 combination with gemcitabine (black bars) and MK-8776 combination with gemcitabine (grey bars) across models for the same quadrant highlighted in each model.
(TIF)

S6 Fig Allogenic T-cell mediated killing of PDXO and cell surface expression of target proteins. a) Schematic of co-culture assay where gastric cancer PDXO GA0091 was labelled with carboxyfluorescein succinimidyl ester (CFSE) used to identify tumor organoids in co-culture. Non-autologous PBMC were stimulated with anti-CD3 and anti-CD28 for three days and activated PBMC were used as effector cells. Co-culture of tumor organoids (target; T) and activated T cell effector (E) cells for 24 or 48 hours at various E:T ratio in 96-well format. b) Organoids co-cultures were dissociated into single cells and stained for live/dead dye. The % of live organoids was quantified using flow cytometry by gating on CFSE^+^ tumor organoid cells for the 2 different incubation periods (24/48hrs) and different E:T ratios. c) Expression of Her2 on the surface of a panel of PDXOs and d) expression of hCD19 engineered into a liver cancer organoid (LI6677-luc PDXO).
(TIF)
