# Supplementary figures and images for: A living biobank of matched pairs of patient-derived xenografts and organoids for cancer pharmacology
Source: PLoS One. 2023 Jan 5;18(1):e0279821. doi: 10.1371/journal.pone.0279821 (PMC9815646; doi:10.1371/journal.pone.0279821)

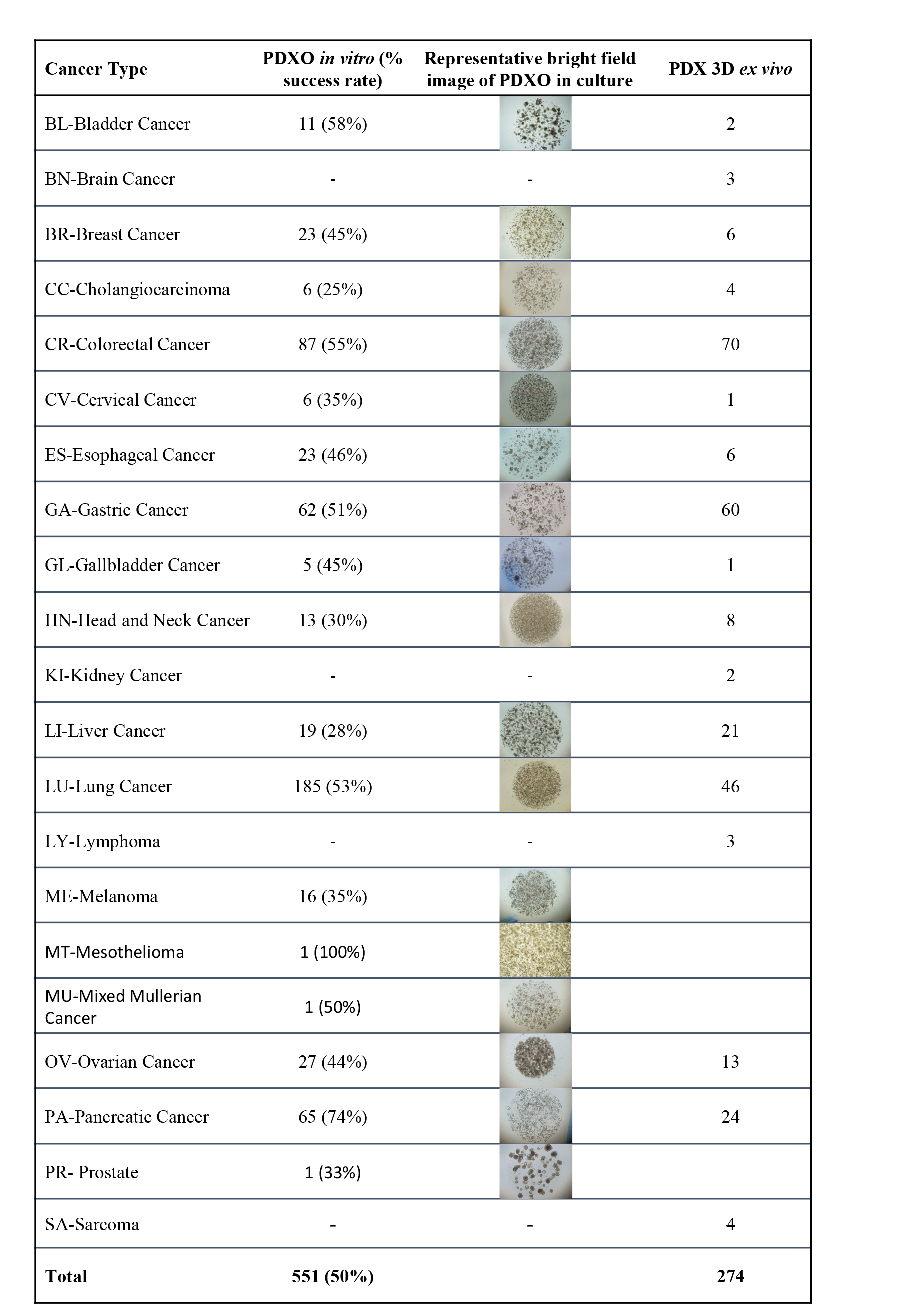

Supplement: S1 Table — For PDXO collections, the % success rate per cancer type was determined based on the number of models successfully biobanked versus the number of models that failed organoid establishment. In comparison the dissociated PDX in 3D ex vivo conditions could not be maintained in continuous culture nor cryopreserved and resuscitated, therefore hindering the generation of a living biobank. PDX ex vivo cultures were approximately 25–35% successful. (TIF) [file pone.0279821.s001.tif]

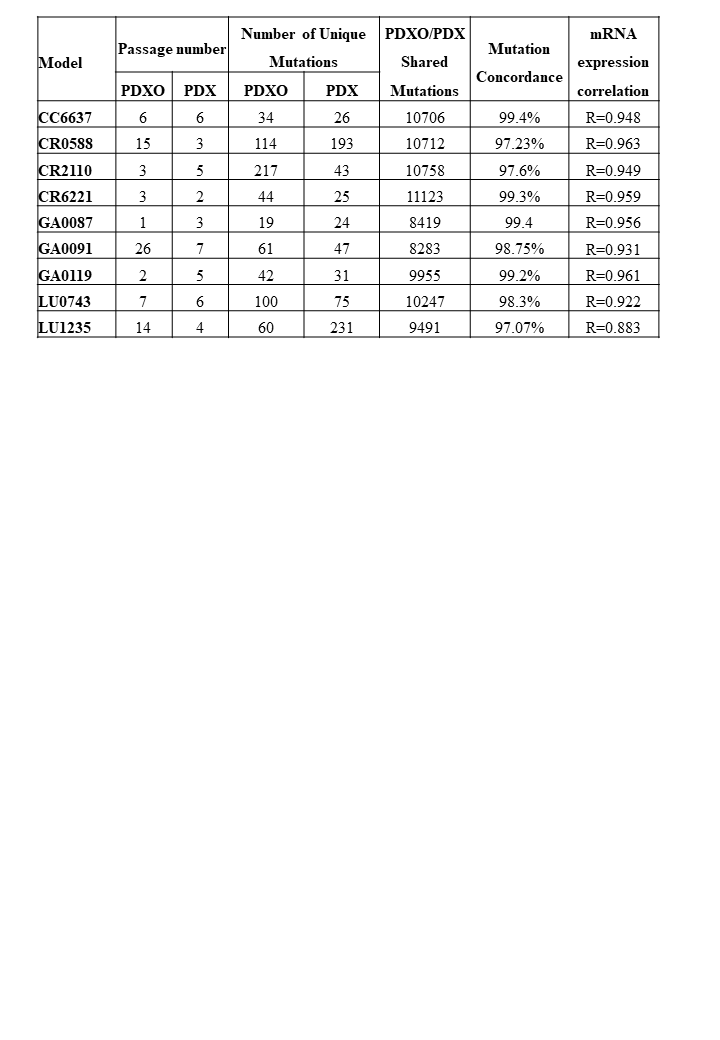

Supplement: S2 Table — (TIF) [file pone.0279821.s002.tif]

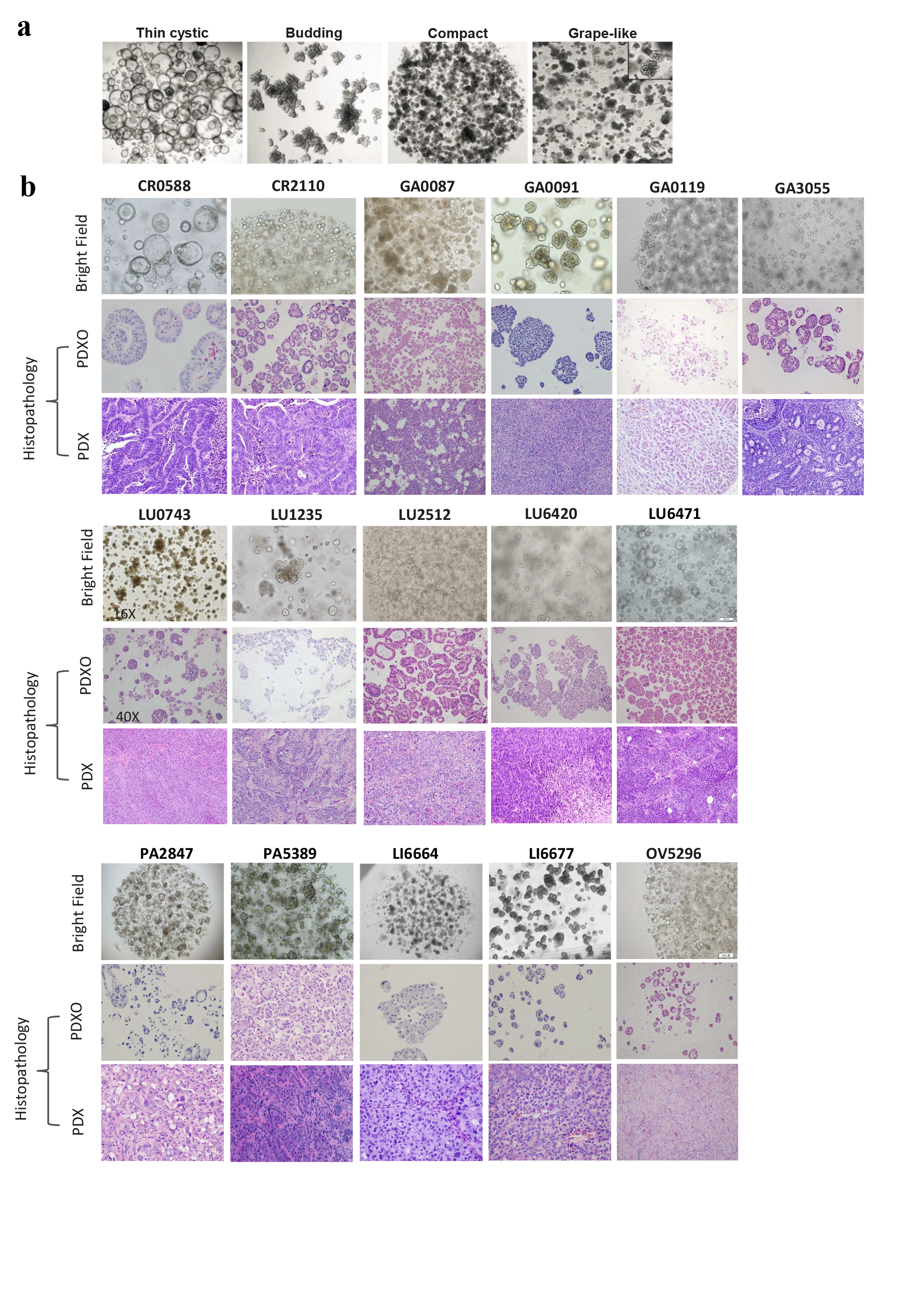

Supplement: S1 Fig — a) Typical cystic, compact, budding and grape-like organoid phenotypes were observed using bright-field microscopy (16x magnification); b) Bright field (top panel) and H&E staining (middle panel, 40x magnification) of PDXO models from different cancer types (colorectal, gastric, lung, pancreatic, liver and ovarian), and H&E staining of matching PDX (bottom panel). (TIF) [file pone.0279821.s003.tif]

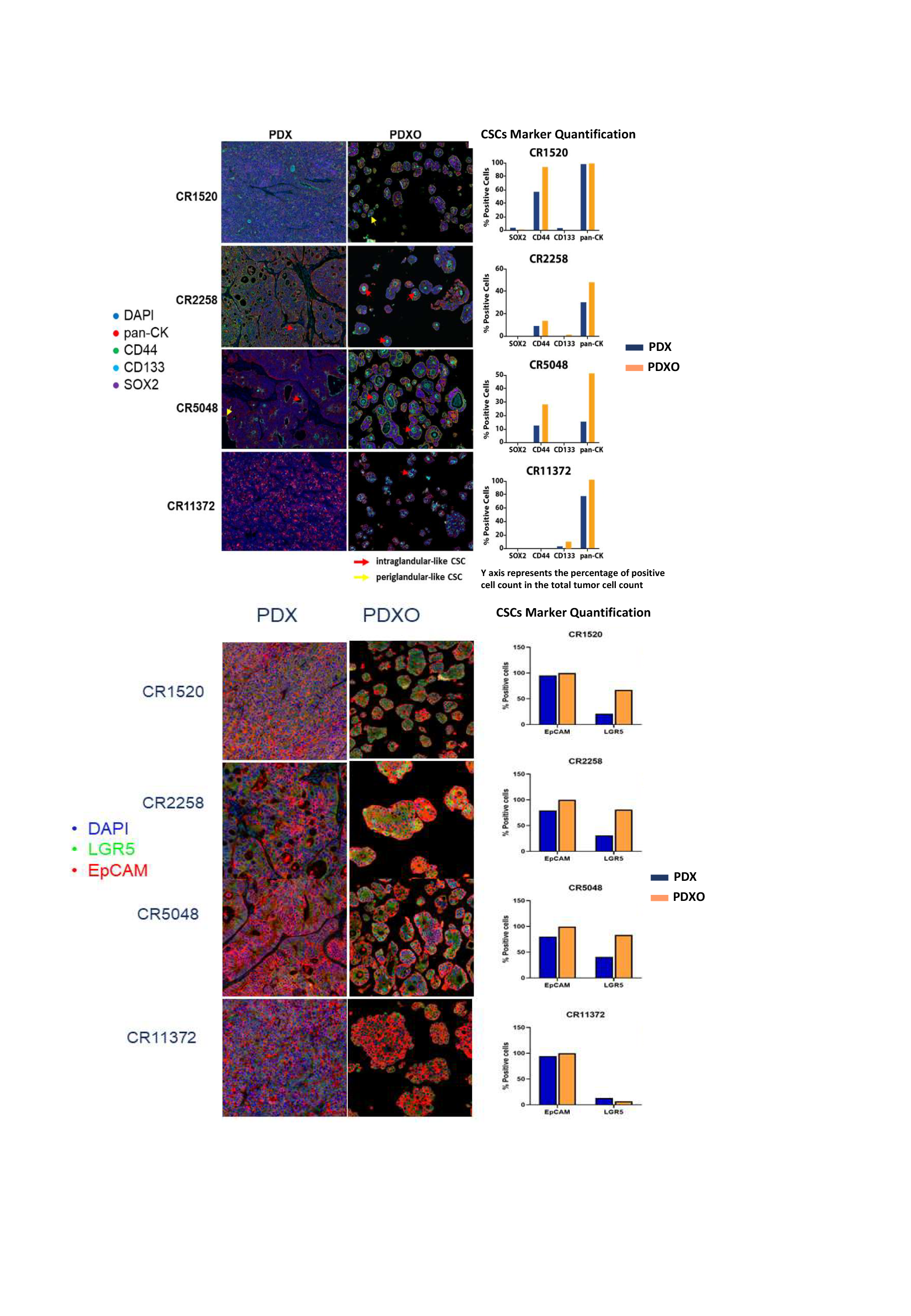

Supplement: S2 Fig — CSC analysis was performed on four CRC-PDX/PDXO pairs (CR1520, CR2258, CR5048, CR11372). Tyramide signal amplification (TSA)-based fluorescent multiplex IHC staining in the Leica Bond Rx automatic platform was performed using a 4-plex/5-color CSC panel: DAPI (Sigma, #9542), CD133 (Cell Signaling, #86781), CD44 (Abcam, ab51037), SOX2 (Cell Signaling, #14962), Lgr5 (Beyotime, #AF1582), EpCAM (Cell Signalling Technology, #14452) and basal cell marker panCK (Abcam, ab270305), followed by whole slide scan using the Vectra® Polaris™ automated quantitative pathology imaging system (PerkinElmer) and quantified by HALO™ image analysis software (Indicalabs) and represented as % of positive cells in the total tumor cell count. Pan-CK and CD44 showed universal co-localization predominantly on the cell surface with broader patterns in corresponding matched models. CD133 displayed a predominantly intraglandular-like staining pattern in PDXOs. Lgr5 expression was high in PDXOs (~100% tumors positive) than PDX, except for CR11372, suggesting more stemness in PDXO. In contrast, SOX2 was broadly deficient in all CRC PDXOs and PDXs. EpCAM showed broader expression in PDXO and consistent with corresponding matched PDX models, which displayed same epithelial-derived pattern in both. (TIF) [file pone.0279821.s004.tif]

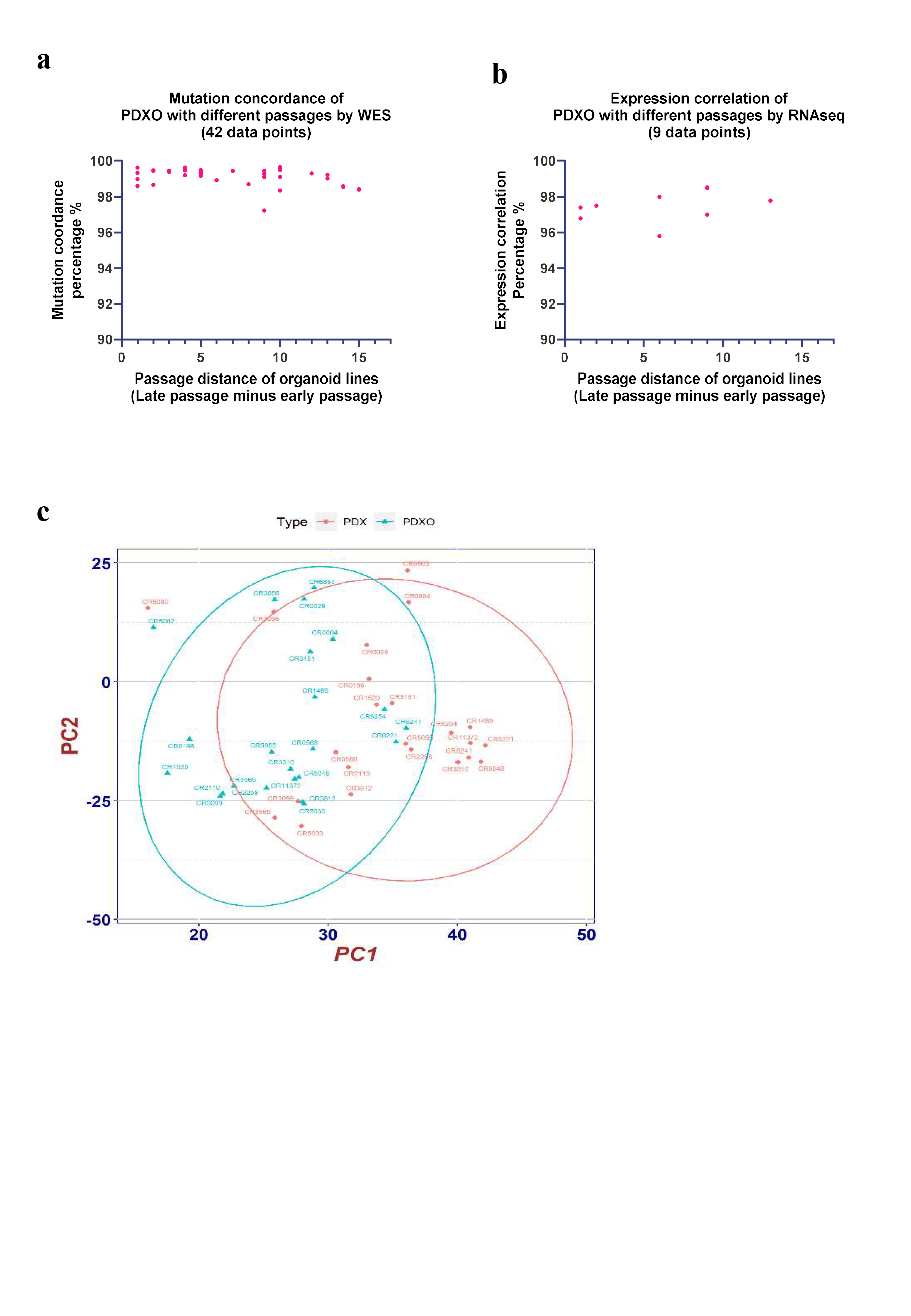

Supplement: S3 Fig — a. Mutation concordance of PDXO across late and early passage analysed by WES; b. Expression correlation analysed by RNA seq. c. For principal component analysis (PCA) of colorectal models we first filtered out low expression genes, then we performed PCA based on the remaining genes. Prior to PCA analysis, gene expression data was normalized by variance stabilization transformations using DESeq2. Finally, we plotted the samples scores of first principle component (PC1) against the sample scores of the second principle component (PC2), while samples derived from PDX/PDXO were grouped into separate eclipses. (TIF) [file pone.0279821.s005.tif]

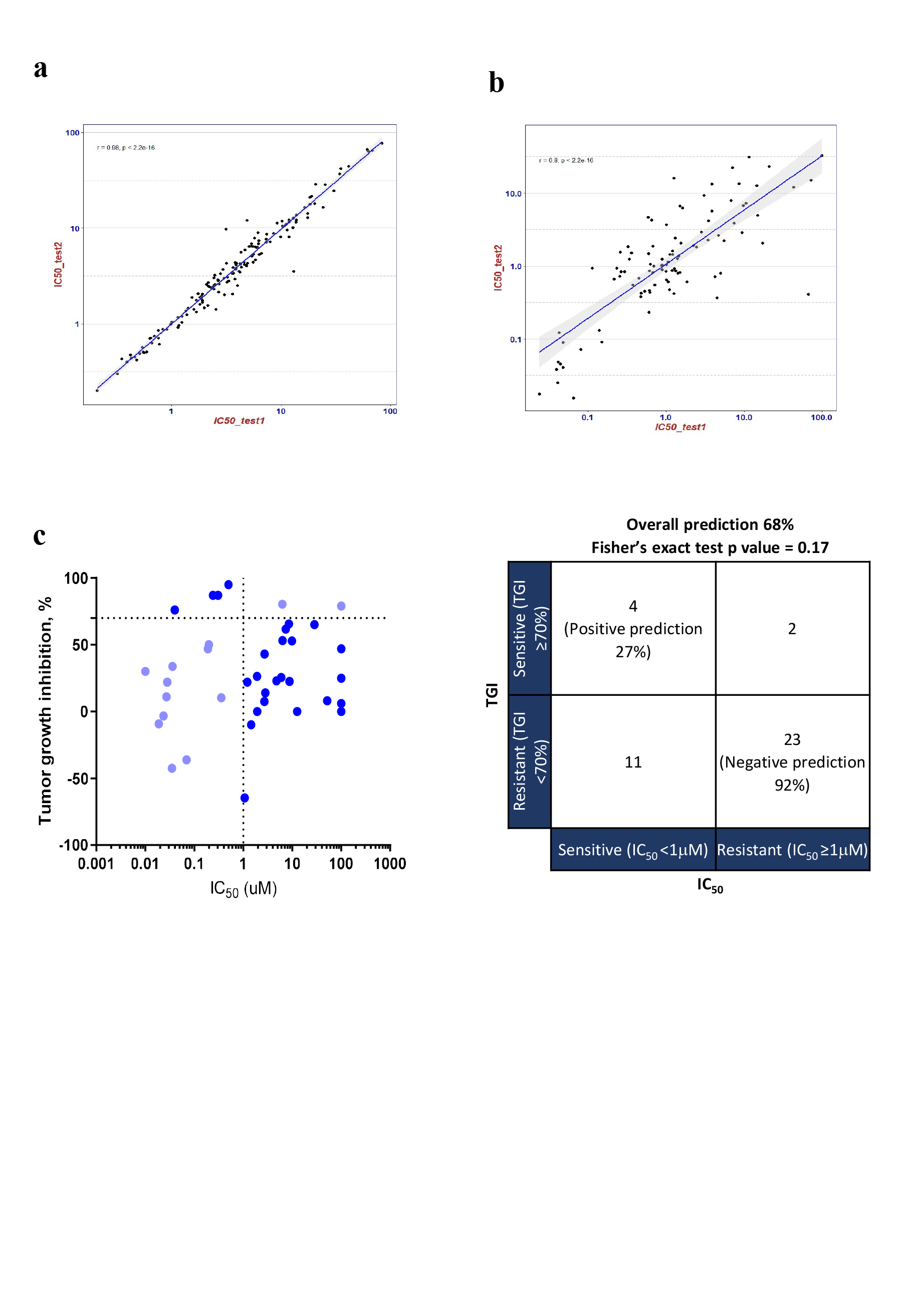

Supplement: S4 Fig — An ex vivo cell bank using cryopreserved ex vivo tumor cells dissociated from freshly isolated PDX tumor tissues. In brief, freshly isolated PDX tumor tissues were digested into single cell suspension using collagenase B. After washing and cell counting, the cells were cryopreserved in liquid nitrogen in standard freezing medium (complete medium supplied with 10% DMSO). Upon compound testing, cryopreserved PDX cells were revived and resuspended in 0.65% methylcellulose (final concentration) and loaded onto 96-well plates and cultured overnight in a 37ºC incubator with a supply of 5% CO2. Test compounds were then added in a 9-point dilution manner in triplicates. Following a 7-day incubation, cell viability was measured by CellTiter-Glo® and plotted to determine IC50 values. Interplate analysis across a) 151 IC50 value datasets from different plates for 2D cell lines or b) 96 IC50 value datasets for PDX ex vivo 3D cultures analyzed for Pearson’s correlation. c) 3D ex vivo assay and in vivo efficacy correlation for PDX models across 40 datasets and 15 different models to determine the predictive power of 3D ex vivo assay system. The criteria for in vitro and in vivo responsive or non-responsive is shown on the right panel with number of datapoints in each category to determine the predictive values and correlation analysis by Fisher’s exact test. (TIF) [file pone.0279821.s006.tif]

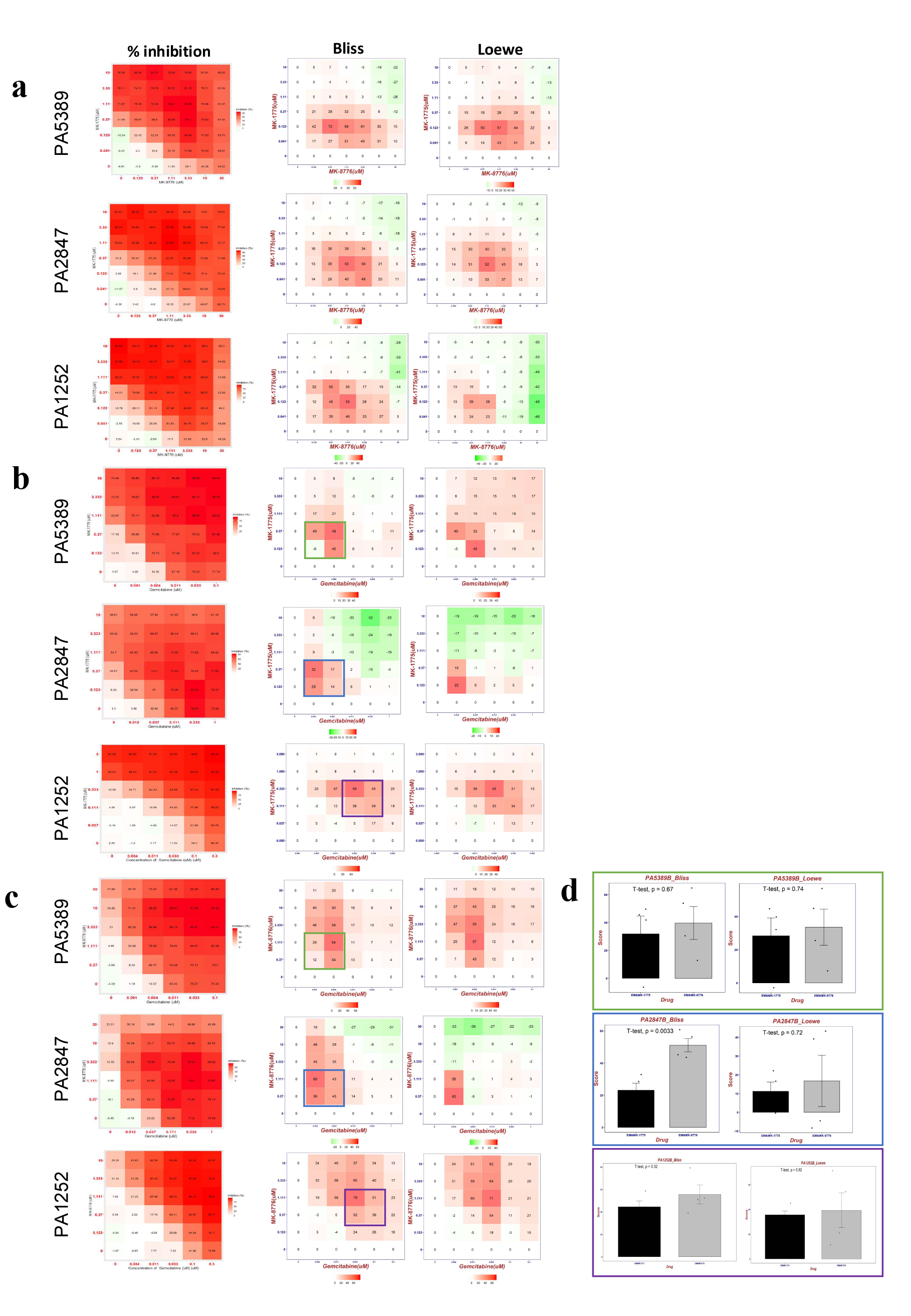

Supplement: S5 Fig — Treatment for 72 hours with a) MK-1775 and MK-8776 combination; b) MK-1775 combination with gemcitabine and c) MK-8776 with gemcitabine represented as % inhibition or organoid death shown as a heat map for each concentration combination in matrix layout (left panel, red represents high, white represents low inhibition) and synergy score calculated using two mathematical models, Bliss (middle) and Loewe (right) and presented as a heat map where red represents synergy and green represents antagonism. d) Comparison of the synergy score for MK-1775 combination with gemcitabine (black bars) and MK-8776 combination with gemcitabine (grey bars) across models for the same quadrant highlighted in each model. (TIF) [file pone.0279821.s007.tif]

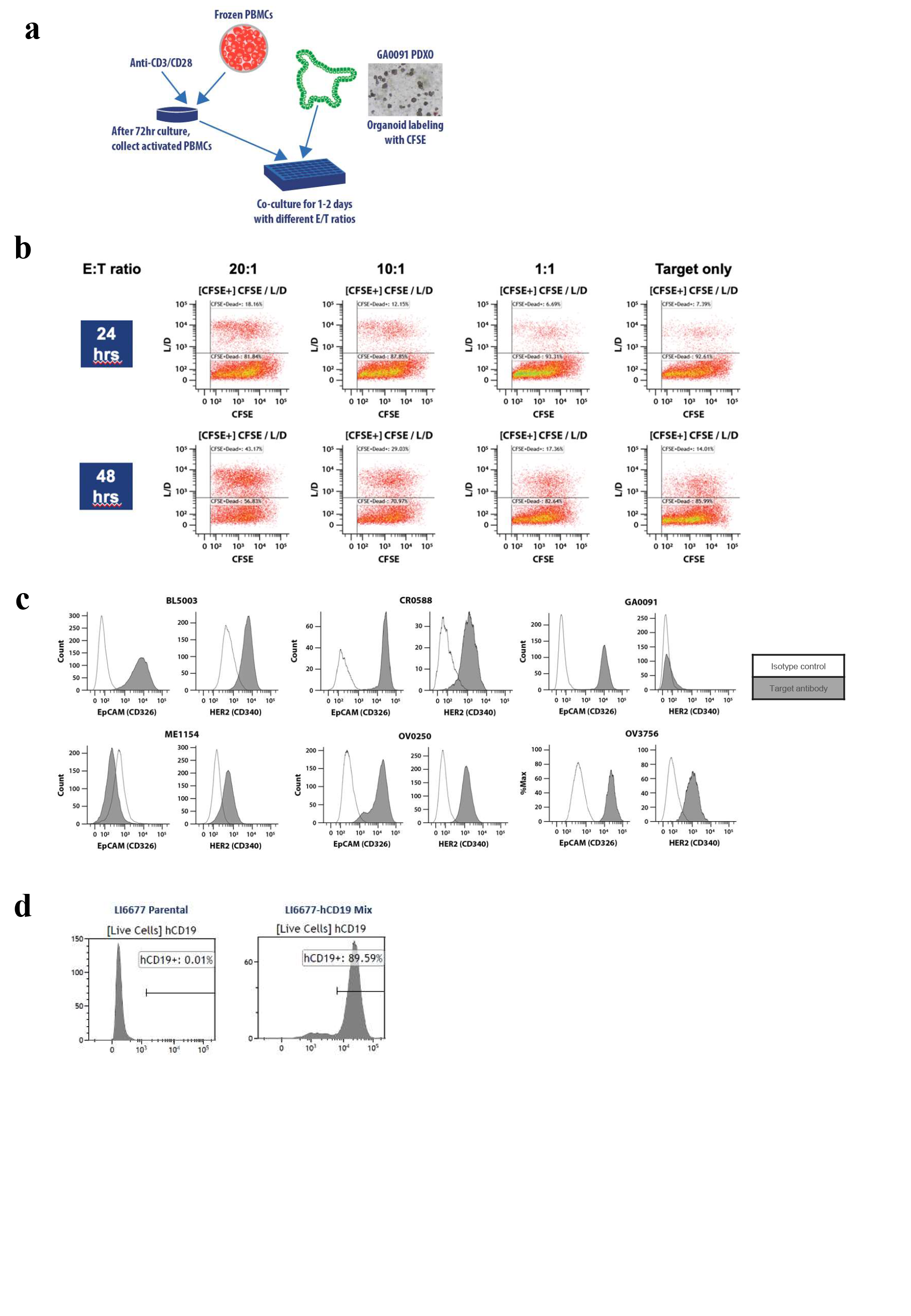

Supplement: S6 Fig — a) Schematic of co-culture assay where gastric cancer PDXO GA0091 was labelled with carboxyfluorescein succinimidyl ester (CFSE) used to identify tumor organoids in co-culture. Non-autologous PBMC were stimulated with anti-CD3 and anti-CD28 for three days and activated PBMC were used as effector cells. Co-culture of tumor organoids (target; T) and activated T cell effector (E) cells for 24 or 48 hours at various E:T ratio in 96-well format. b) Organoids co-cultures were dissociated into single cells and stained for live/dead dye. The % of live organoids was quantified using flow cytometry by gating on CFSE+ tumor organoid cells for the 2 different incubation periods (24/48hrs) and different E:T ratios. c) Expression of Her2 on the surface of a panel of PDXOs and d) expression of hCD19 engineered into a liver cancer organoid (LI6677-luc PDXO). (TIF) [file pone.0279821.s008.tif]
